# Digestibility of Bovine Serum Albumin and Peptidomics of the Digests: Effect of Glycation Derived from α-Dicarbonyl Compounds

**DOI:** 10.3390/molecules23040712

**Published:** 2018-03-21

**Authors:** Bulei Sheng, Lotte Bach Larsen, Thao T. Le, Di Zhao

**Affiliations:** 1 College of Food Science and Engineering, South China University of Technology, 381 Wushan Road, Guangzhou 510640, China; shengbulei@outlook.com; 2 Department of Food Science, Aarhus University, Blichers Allé 20, 8830 Tjele, Denmark; lbl@food.au.dk (L.B.L.); thaothithu.le@ecu.au (T.T.L.)

**Keywords:** α-dicarbonyl compounds, glycation, advanced glycation end products, digestibility, bovine serum albumin

## Abstract

α-Dicarbonyl compounds, which are widely generated during sugar fragmentation and oil oxidation, are important precursors of advanced glycation end products (AGEs). In this study, the effect of glycation derived from glyoxal (GO), methylglyoxal (MGO) and diacetyl (DA) on the in vitro digestibility of bovine serum albumin (BSA) was investigated. Glycation from α-dicarbonyl compounds reduced digestibility of BSA in both gastric and intestinal stage of digestion according to measurement of degree of hydrolysis. Changes in peptide composition of digests induced by glycation were displayed, showing absence of peptides, occurrence of new peptides and formation of peptide-AGEs, based on the results obtained using liquid chromatography electron-spray-ionization tandem mass spectrometry (LC-ESI-MS/MS). Crosslinked glycation structures derived from DA largely reduced the sensitivity of glycated BSA towards digestive proteases based on sodium dodecyl sulfate polyacrylamide gel electrophoresis (SDS-PAGE) results. Network structures were found to remain in the digests of glycated samples by transmission electron microscope (TEM), thus the impact of AGEs in unabsorbed digests on the gut flora should be an interest for further studies.

## 1. Introduction

α-Dicarbonyl compounds are compounds with two adjacent carbonyl groups, commonly being generated during caramelization or in the intermediate stage of the Maillard reaction. 3-Deoxyglucosone (3-DG, C-6 backbone), glyoxal (GO), methylglyoxal (MGO) and diacetyl (DA) are the major α-dicarbonyl compounds derived from sugar fragmentation [1,2]. Oil oxidation is another important source of these compounds [3].

These α-dicarbonyl compounds are widely detected in commonly consumed foods, such as sweets, honey, dairy, jams and bakery products, and their contents increase during heat treatment of food processing [4]. For example, GO content was reported to increase from less than 0.3 mg/L to approximately 2.5 mg/L after ultra-high temperature (UHT) treatment of Milk [5]. Heating safflower oil, cheese, butter or margarine (at 100 and 200 °C for 1 h) resulted in increased DA content ranging from 13.9 to 2835.7 mg/kg [3,6]. These studies show the substantial formation of α-dicarbonyl compounds in food upon processing and storage. Therefore, the nutritional and health significance of these compounds require further attention and research.

After being generated in food processing or storage, these compounds can react with the side chains of Lys and Arg residues of dietary proteins, generating advanced glycation end products (AGEs). GO is the precursor of N (ε)-carboxymethyllysine (CML), GO hydroimidazolone (G-H) and glyoxal lysine dimer (GOLD), whereas MGO is the precursor of N(ε)-carboxyethyllysine (CEL), MGO hydroimidazolone (MG-H) and methylglyoxal lysine dimer (MOLD) [7,8]. In addition, reaction between DA and side chain of Arg residue of a protein was reported to produce hydroimidazolone compounds, which may be related to the etiology of obliterative bronchiolitis [9]. AGEs, formed in vivo or endogenous AGEs, are involved in the pathology of diabetes, cardiovascular disease and cataracts [10,11,12]. AGEs present in food (dietary AGEs) are important contributors to the endogenous AGEs pool [13]. According to several animal and clinical studies, the absorbed dietary AGEs are a potential hazard after being absorbed in small intestine, because of their ability to combine with their receptors (RAGEs) in vivo [14,15].

Dietary AGEs can be in free state, peptide-AGEs and protein-AGEs, and protein-AGEs are the most common origin of dietary AGEs. Before reaching the small intestine, protein-AGEs are enzymatically hydrolyzed into absorbable or unabsorbable fractions during their passage through the gastrointestinal tract. Therefore, digestion of protein-AGEs largely determines the absorption of AGEs in digests by small intestine, and was studied in this work. Considering the ubiquity of α-dicarbonyl compounds in processed food and their role in AGEs formation, investigating the effect of glycation derived from α-carbonyl compounds on protein digestibility and analyzing the digests of protein-AGEs should help to elucidate the nutritional impact of these α-carbonyl compounds and the health effect of protein-AGEs.

Subsequently, bovine serum albumin (BSA), a well-studied protein, was selected as the substrate for glycation and digestion. GO, MGO and DA, three kinds of major α-carbonyl compounds, were selected as glycation reagents to prepare protein-AGEs. In addition to analyzing the degree of hydrolysis (DH), we also studied the peptidomics in digests using LC-ESI-MS/MS and Mascot database, which was reported in only a few studies [16,17]. Identifying the peptide and AGEs in digests could provide useful information for further studying the nutrition of glycated protein and the absorptivity of protein-AGEs.

## 2. Results and Discussion

### 2.1. Glycation During Incubation

#### 2.1.1. Loss of Primary Amino Group (−NH_2_)

ε-NH_2_ and guanidino group of Lys and Arg residues are considered as the major glycation sites of protein [18]. Loss of primary amino group (−NH_2_) in protein after 2 h of heating are shown in Table 1, which indicate that the activities of the α-dicarbonyl compounds decrease according to: GO > MGO > DA. The electron donating and steric hindrance of the methyl groups beside the carbonyl carbons in MGO and DA, as described by Meade et al., could be reason for their lower activity than GO [19].

#### 2.1.2. Changes in Aggregates Conformation

**Figure 1:**
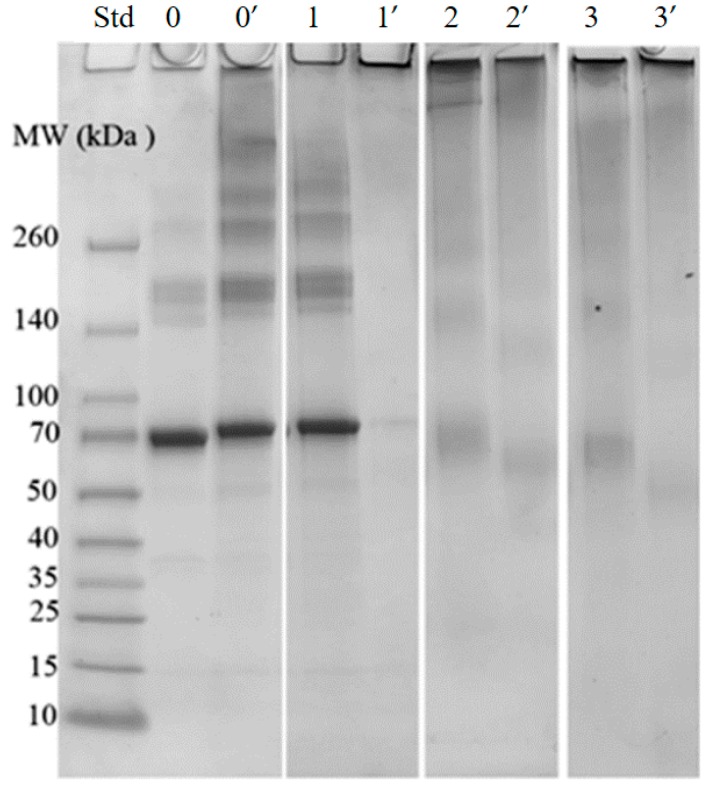
SDS-PAGE of control and glycated BSA after 2 h of incubation. Lane 0 refers to control BSA. Lanes 1, 2, and 3 refer to BSA glycated with 10 mM of GO, MGO and DA, respectively. Lanes 0, 1, 2 and 3 represent reduced (with DTE) samples, Lanes 0’, 1’, 2’ and 3’ represent non-reduced samples.

**Figure 2:**
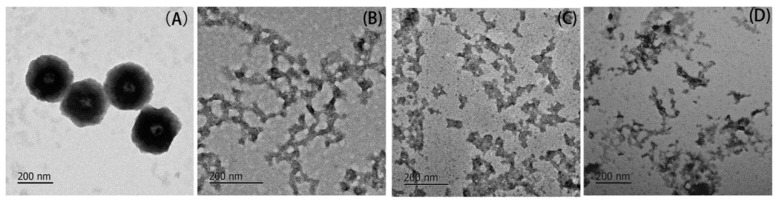
Representative TEM images of both: control BSA (**A**); and BSA glycated with 10 mM: GO (**B**); MGO (**C**); and DA (**D**) after 2 h of incubation at 95 °C.

**Figure 3:**
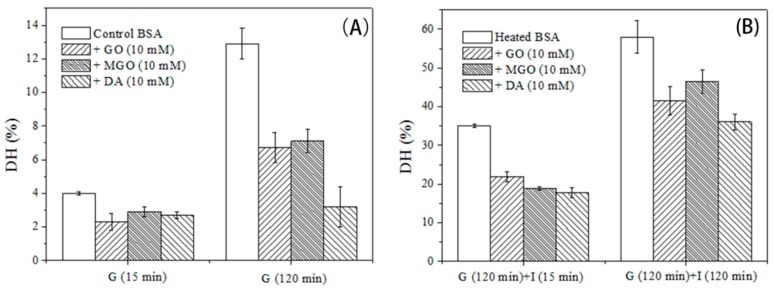
DH of the control and glycated BSA during in vitro gastric (**A**) and subsequent intestinal (**B**) digestion. G (15 min) and G (120 min) refer to samples digested for 15 and 120 min in gastric stage, respectively. I (15 min) and I (120 min) represent samples digested for 15 and 120 min digestion in intestinal stage, respectively. Data are an average of triplicate data from two independent assays, and the error bars refer to standard deviations.

Cys (121) of BSA can participate in the formation of intermolecular disulfide bond, which can be disconnected by DTE. Glycation generates crosslinked structures, including pentosidine, GOLD and MOLD which also connect protein chains, as revealed in previous studies [7,8]. Figure 1 shows the SDS-PAGE of both reduced (by DTE) and non-reduced protein aggregates, aiming to distinguish intermolecular disulfide bonds from crosslinked glycation structures. Control BSA aggregates (Figure 1, lane 0’) are largely dissociated to monomers or dimers (Figure 1, lane 0) by DTE. Comparatively, glycated BSA aggregates (Figure 1, lane 1’, 2’ and 3’) are less sensitive to DTE and largely remained in stacking gel (Figure 1, Figure 2 and Figure 3). These findings suggest that the disulfide bonds are dominant in control BSA, while crosslinked glycation structures are more pronounced in glycated BSA.

Representative images of the protein aggregates after 2 h of heating are shown in Figure 2. Control BSA (Figure 2A) is found to aggregate into globular conformation (approximate 200 nm in diameter), whereas totally different conformation of glycated aggregates (Figure 2B–D) are observed. All the glycated aggregates appear to be in uncompact and network conformations. Analogous TEM micrographs were obtained in the BSA samples co-incubated with 50 mM glyoxylic acid-treated for 83 weeks of incubation at 37 °C [20]. The refolding behavior of BSA should be limited by glycation through increasing the steric hindrance and electrostatic interaction, as reported in the studies of Pinto and Liu et al. [21,22]. 

### 2.2. Changes in Digestibility

#### 2.2.1. Change in Degree of Hydrolysis (DH) during Gastrointestinal Digestion

Digestibility of a protein is associated both with its amino composition and higher level structures. Glycation are shown to modify both Lys residues as well as change aggregation behavior. Therefore, the digestibility of glycated samples should be changed by glycation, and this is addressed in the following sections.

Figure 3 illustrates changes in the DH during gastrointestinal digestion. Glycation has been reported to modify the Lys and Arg residues, blocking the action of trypsin in intestinal digestion [23,24]. However, few studies have investigated the influence of glycation on digestion in the gastric stage. Notably, decreases in the DH of glycated samples during the gastric digestion are found in this work. For example, an around 13% DH value is found in the control BSA sample, compared to 3–7% DH in the glycated BSA after 120 min of gastric digestion. In our previous study, the digestibility of GO-glycated β-casein and β-lactoglobulin decreased in smaller degree than GO-glycated BSA in this work [16]. This discrepancy may be possibly attributed to the lower level of active site (Lys and Arg residuals) in β-casein (7.2%) and β-lactoglobulin (11.2%) compared with BSA (14.1%). Pepsin preferentially acts on Phe, Tyr and Trp residues, that are not affected by glycation [25]. Therefore, glycation decrease the DH value in gastric digestion most likely by changing protein structures other than blocking the enzymatically cleavage sites. Increases in steric hindrance and electrostatic interactions induced by glycation may not only impede the aggregation process, but also hinder the interactions between pepsin and glycated substrates [21,22]. Regarding the successive intestinal digestion, glycation further lead to declined DH in glycated BSA. For example, DH value in control BSA increases by 45% in intestinal digestion, which is compared to the 30%–38% increases in DH values of glycated BSA in the same process. It should be noted that DA-derived glycation result in largest decrease in DH value even though the lowest activity of DA among the three studied α-dicarbonyl compounds in glycation. This result can be related with the biggest molecular skeleton of DA among the selected α-dicarbonyls, which may cause larger steric hindrance and hinder the action of proteases to the largest extent.

#### 2.2.2. Dynamic Digestion Process Evaluated by SDS-PAGE

**Figure 4:**
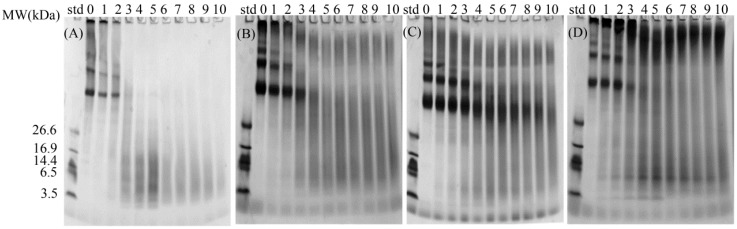
SDS-PAGE of digests from: control BSA (**A**); and BSA glycated with 10 mM: GO (**B**); MGO (**C**); and DA (**D**). Lane 0 indicates undigested samples. Lanes 1, 2, 3, 4 and 5 indicate samples digested for 1, 5, 15, 60 or 120 min in gastric stage, respectively. Lanes 6, 7, 8, 9 and 10 indicate samples digested for 120 min in gastric stage followed by 1, 5, 15, 60 or 120 min digestion in intestinal stage, respectively.

Figure 4 illustrates the electrophoretogram of the digests during the gastrointestinal digestion process. Glycated aggregates in stacking gel can still be observed after the gastrointestinal digestion in lane 10 of each gel, while control aggregates disappear after 60 min of gastric digestion. This result suggest that aggregates induced by heating may have minor hindrance to the digestibility than aggregates induced by glycation. Compared with GO- and MGO-glycated samples (Figure 4B,C), aggregates in DA-glycated sample (Figure 4D) appears to be less sensitive to enzymatic hydrolysis and largely remained in stacking gel after the complete gastrointestinal digestion. This finding is in line with the lowest DH value of DA-glycated BSA, as shown in Figure 3. The stronger resistance of DA-glycated sample to digestive proteolysis may be related with the DA-derived crosslinked glycation structures which may own bigger molecular skeleton than those deriving from GO and MGO. Indeed, the intermolecular crosslinked structures had been reported to particularly reduce the accessibility of potential cleavage sites in the study of Pinto et al., and this phenomenon was reconfirmed in this study [24].

**Figure 5:**
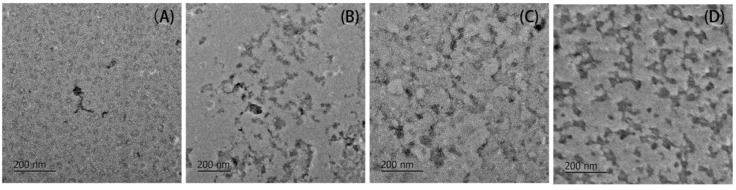
Representative TEM images of gastrointestinal digests from: control BSA (**A**); and BSA glycated with 10 mM: GO (**B**); MGO (**C**); and DA (**D**).

Due to the decreased digestibility shown by DH and the SDS-PAGE, more digests with larger sizes are found in the glycated samples (Figure 5B–D). Only a few small fibrils are found in the gastrointestinal digests of the control BSA. Contrarily, network structures are shown to remain in each digest of glycated BSA. Since free amino acids and di- and tripeptides are common targets for carriers in intestinal absorption, smaller peptides after gastrointestinal digestion are more likely to be absorbed after passing the epithelial mucus layer and being hydrolyzed to absorbable products by peptidases [26]. Therefore, TEM images for digests of glycated samples clearly imply the nutritional loss of BSA induced by glycation derived from α-dicarbonyls. Bui et al. and Hellwig et al. reported utilization of MRPs, such as the Amadori products, CML, pyrraline and maltosine, by the gut flora to various degrees [27,28]. MRPs in digests were also found to change the balance of the gut flora in different ways, depending on the differences in proteins, reducing sugars and heat treatments [29,30,31]. Therefore, the impact of AGEs present in unabsorbable fractions on the gut flora should be considered.

### 2.3. Peptides Analysis in Digests

#### 2.3.1. Changes in Peptidomics of Digests

**Figure 6:**
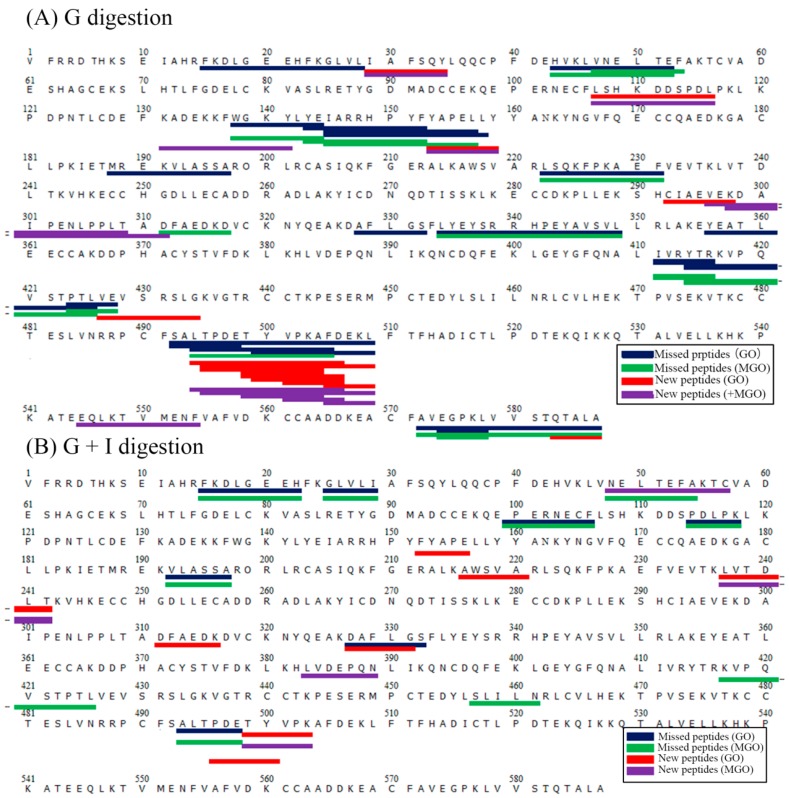
Gastric (**A**); and gastrointestinal (**B**) digested peptides of BSA changed by glycation derived from 1 mM GO or MGO for 2 h. Peptides in blue and green represent the missed digested peptides identified in GO- and MGO-glycated samples compared with control sample, respectively; peptides in red and purple represent new digested peptides identified in GO- and MGO-glycated samples compared with control sample, respectively.

Figure 6 provides a comparison of the peptides released from glycated (with 1 mM GO or MGO) and control BSA after gastric (Figure 6A) and complete gastrointestinal digestion (Figure 6B), as identified using LC-ESI-MS/MS. Even being glycated with 1 mM of α-dicarbonyl compounds, different peptidomics profiles between control and glycated samples are found, including both the absence of some peptides (not detected in glycated samples, in blue or green color) and the occurrence of new peptides (not detected in control samples, in red and purple color). After gastric digestion, 17 and 14 missed peptides are identified, whereas 14 and 11 new peptides are detected in the gastric digests of BSA glycated with 1 mM GO and MGO, respectively. These changes in peptidomics further confirm the influence of glycation structures on the action of pepsin. Figure 6B illustrates the peptidomics profiles after the whole gastrointestinal digestion. Seven and ten missed peptides are identified, whereas seven and four new peptides are detected compared with control in the gastrointestinal digests of GO- and MGO-glycated BSA, respectively. These results clearly show the changes in peptidomics for digests of glycated sample, which reflect that the action preference and efficient of digestive proteases may be changed by glycation derived from α-dicarbonyl compounds even though more studies are needed.

#### 2.3.2. Peptide-AGEs in Digests

Table 2 illustrates the eight gastric and six gastrointestinal digested peptides-AGEs (CML, CEL, G-H1 and MG-H1), which could indicate the influence of these structures on the digestibility of protein. These sequences are found with no glycation structures at the C-terminus, and this is in line with consensus that glycation block the action of trypsin [23,24]. CML, G-H1 or MG-H1 are found to locate in the positions next to the C-terminus of sequences 2, 4, 5, 6, 7, 13 and 14. This result could imply that these glycation structures will not stop the action of related digestive protease. Non-crosslinked glycation structures, such as Amadori products, N(ε)-carboxymethyllysine (CML) and GO hydroimidazolone (G-H), change only the side chain of Lys and Arg. Comparatively, crosslinked structures including pentosidine, GOLD and MOLD can connect different protein chains together, thus may limit the flexibility of protein and result in higher hindrance to the proteolysis. Peptide-AGEs have been proved to be absorbed more efficiently by Caco-2 Cell than their free form (Hellwig et al., 2009; Hellwig et al., 2011) [32,33]. Therefore, the absorption and in vivo bioactivity of these peptide-AGEs by intestinal epithelial cell requires to be further investigated.

Residues near to the protease cleavage sites were reported to largely change the hydrolysis kinetics of a protease, which is termed a “secondary enzyme-substrate interaction” [34]. This phenomenon was reported for a variety of proteases, including pepsin, trypsin, chymotrypsin and elastase [35,36]. Analogously, in this study, crosslinked and non-crosslinked glycation structures near to potential cleavage site may also change the action preference and hydrolysis kinetics of pepsin, trypsin, chymotrypsin and elastase, resulting in discrepancies in the digested peptides in Figure 6. 

## 3. Materials and Methods 

### 3.1. Materials

Bovine BSA (≥98%), GO (40% aqueous solution), MGO (40% aqueous solution), DA (analytical standard) pepsin (from porcine, ≥250 unit/mg) and pancreatin (from porcine, 8× USP), used for the simulated gastrointestinal digestion, were purchased from Sigma-Aldrich (Steinheim, Germany).

### 3.2. Glycation Model

Glycation was performed in phosphate buffer (50 mM, pH 7) containing 5 mg/mL of protein and 1 or 10 mM (1.62 fold of active sites) GO, MGO and DA. These mixtures were heated in a water bath at 95 ± 1 °C in 10 mL sealed glass vials to simulate heat treatment during food processing. BSA were heated independently as control samples. After incubation, the protein mixtures were dialyzed at 4 °C for 1 day using dialysis tubes with a 3-kDa molecular weight cut-off to ensure the removal of excess carbonyls and salts. The dialyzed solutions were lyophilized and stored at −20 °C prior to the digestion assay. 

### 3.3. In Vitro Digestion

A static in vitro digestion system was applied according to Minekus et al. with some adjustments in the dosage of enzymes [37]. Considering the relative low concentration (5 mg/mL) of digested substrate, the dosage of enzymes was lowered in this work. Activity of pepsin and pancreatin were identified as 6106 units/mg and 32 *p*-Toluene-Sulfonyl-l-arginine methyl ester (TAME) units/mg, according to the supplementary document in the study of Minekus et al. A lyophilized protein (20 mg) was redissolved in 4 mL of simulated gastric fluid (SGF, freshly prepared according to the reference). Then, pepsin (9 mg/mL) was added to obtain a final activity of 500 units/mL. Gastric digestion was maintained at 37 °C for 120 min, and the reaction was stopped by elevating the pH to 7.0 with 3 mL of simulated intestinal fluid (SIF, prepared according to the reference). At each set time point of 1, 5, 15, 60 and 120 min, 200 μL of digested sample was withdrawn, immediately mixed with 200 μL of SIF, frozen with liquid nitrogen and stored at −20 °C until further analysis.

In the successive intestinal digestion, pancreatin was added to the solution after the addition of the SIF to give a final activity of 5 TAME U/mL. This mixture was incubated at 37 °C for 120 min, and the reaction was stopped by heating at 100 °C for 3 min. At each time point corresponding to 1, 5, 15, 60 and 120 min, 300 μL of digested samples was withdrawn, heated at 100 °C for 3 min, frozen in liquid nitrogen and stored at −20 °C until further analysis.

### 3.4. Fluorescamine Assay

#### 3.4.1. Loss of −NH_2_ during Glycation

The loss of −NH_2_ was measured using a modified method of Yaylayan et al. [38]. A heated sample (30 µL) was mixed with 900 µL of 0.2 M sodium tetraborate buffer (pH 8.5), then 300 µL of 0.2 mg/mL of fluorescamine in dry acetone was added. Fluorescence was measured using excitation and emission wavelengths of 390 and 480 nm, respectively, in a Synergy 2 Multi-Mode microplate reader (Holm & Halby, Brøndby, Denmark). The −NH_2_ content was expressed as a relative amount, assuming that 100% was equal to the −NH_2_ content of control sample before heating.

#### 3.4.2. DH of Digests

Another fluorescamine assay was conducted to determine the DH of digested protein aggregates strictly based on method of Petrat-Melin et al. [39]. Digested sample (75 μL) was mixed with 75 μL of 24% TCA and precipitated on ice for 30 min. Then, the solution was centrifuged at 13,000 rpm for 20 min at 4 °C. After that, 30 μL standard (L-leucine) or sample supernatant was withdrawn and mixed with 900 μL sodium tetraborate (0.1 M, pH 8.0). Then, 300 μL of fluorescamine acetone solution (0.2 mg/mL) was added to the above solution before reading the Fluorescence was measured using excitation and emission wavelengths of 390 and 480 nm, respectively. The DH was calculated as follows:

(1) $$\mathrm{DH}=\frac{\left[-{\mathrm{NH}}_{2}\;\left(h\right)\right]-\left[-{\mathrm{NH}}_{2}\;\left(0\right)\right]}{\left[-{\mathrm{NH}}_{2}\;\left(\infty \right)]-[-{\mathrm{NH}}_{2}\;\left(0\right)\right]}$$

where [−NH_2_] is equal to the concentration of primary amines in the hydrolyzed (h) or unhydrolyzed (0) sample, and [−NH_2_ (∞)] is equal to the theoretical maximal primary amine concentration, assuming total digestion to free amino acids. [−NH_2_ (∞)] was calculated as follows:

(2) $$\left[-{\mathrm{NH}}_{2}\;\left(\infty \right)\right]=\frac{\left[1+f\;\left(\mathrm{Lys}\right)\right]C}{\mathrm{MW}\left(\mathrm{AA}\right)}$$

where f (Lys) is the relative fraction of lysine residues in the protein, C is the protein concentration, and MW (AA) is the mean molecular weight of amino acids in the protein.

### 3.5. Sodium Dodecyl Sulfate Polyacrylamide Gel Electrophoresis (SDS-PAGE)

#### 3.5.1. SDS-PAGE of Aggregates

Precast gels (4–15%, Bio-Rad, Hercules, CA, USA) were applied for the detection of aggregation of substrate proteins during glycation based on the method of Laemmli [40]. The stored samples (10 mg/mL) were diluted 10 folds with Laemmli buffer (20 mM Tris, 2% SDS, 20% glycerol, pyronin Y, Steinheim, Germany) with or without addition of 0.02 M dithioerythritol (DTE, Steinheim, Germany). After being heated at 90 °C for 5 min, each diluted samples (10 µL) was loaded on individual well and electrophoresed at 200 V for around 45 min prior to staining with Coomassie Blue G 250. Images were scanned by a Molecular Imager (ChemiDocTM XRS+, Bio-Rad, Philadelphia, PA, USA).

#### 3.5.2. SDS-PAGE of Digests

Digested samples were electrophoresed by SDS-PAGE on a Mini Protean II system (Bio-Rad Laboratories, Richmond, CA, USA) using the method of Schagger et al. [41]. Precast gels (NovexTM, 10–20%, Thermo Fisher, Waltham, USA) were conducted to analyze the dynamic proteolysis of proteins during digestion. The digested samples were diluted 5 folds with sample buffer (containing 0.2 M Tris, 0.2% SDS, 4% glycerol and 0.05% Coomassie G250, Steinheim, Germany) and heated at 90 °C for 5 min. The diluted samples (~20 μg) were loaded onto a precast gel and run at 125 V before staining with Coomassie G250. 

### 3.6. Transmission Electron Microscopy (TEM)

The conformations of the protein aggregates and their digests were observed by TEM. Samples (10 μL, 0.1 mg/mL) were transferred to carbon-stabilized grids for 30 s. Excess sample was removed with ashless filter paper. Then, the grids were stained with 0.5% phosphotungstic acid (PTA) for 30 s. After removing the remaining stain, the grids were left to air dry for 30 min before being examined and photographed using TEM (2100F, JEOL, Tokyo, Japan) at an accelerating voltage of 200 kV.

### 3.7. LC-ESI-MS/MS

Digested peptides after both gastric and gastrointestinal digestion were identified by LC-ESI-MS/MS using the method of Le et al. [42]. Digested samples from the control and glycated samples were mixed with 0.2% formic acid in a 1:1 ratio prior to filtration through a 10-kDa cut-off filter (14,000× *g*, 4 °C for 15 min). Then, 10 µL solution was loaded onto an Aeris Peptide C18 column (250 mm × 2.1 mm, 3.6 μm, Phenomenex, Torrance, CA, USA) in an Agilent LC 1200 directly connected to an HCT Ultra Ion Trap (Bruker Daltonics, DE, USA). Solvent A was 0.1% formic acid, and solvent B was 90% acetonitrile with 0.1% formic acid. A gradient run was as follows: 2% B for 5 min, 40% B for 80 min, 80% B for 20 min and 2% B for 5 min. The MS was scanned from 400 to 1800 *m*/*z*, and MS/MS was scanned from 150 to 1800 *m*/*z*.

The MS/MS spectra were recorded and analyzed using Data Analysis and Biotools software (Bruker Daltonics, DE, USA). The obtained tandem mass spectra were searched against a custom database (Mascot v2.4, Matrix Science, London, UK) according to Rauh et al. with some adjustment in the choice of modification [43]. The following search parameters were applied: protease: unspecific; mass tolerance for precursor ions: 15 ppm; mass tolerance for production ions: 0.6 Da; variable modifications: CML and G-H1 modification on Lys or Arg for the digests of samples glycated with GO, and CEL and MG-H1 modification on Lys or Arg for the digests of samples glycated with MGO. Only peptides identified as significant (expect *p* < 0.05) were considered.

## 4. Conclusions

The effects of glycation derived from α-dicarbonyl compounds on protein digestibility were studied in a model system with GO MGO and DA. In addition to blocking tryptic cleavage sites, glycation also reduced gastric digestibility, which indicates the hindrance of glycation structures on the action of pepsin. Compared with glycation derived from GO and MGO, DA-derived glycation was evidenced to result in larger hindrance to enzymatic hydrolysis, possibly due to stronger hindrance of crosslinked structure derived from DA. In addition, glycation substantially changed the peptidomics of digests, which may further alter the absorption of these peptides in small intestine. Furthermore, the impact of MRPs in unabsorbed digests on the gut flora should be an interest for further studies.

## Figures and Tables

**Table 1:** Loss of −NH_2_ after 2 h of heating at 95 °C.

Sample ID	Loss of −NH_2_ (%)
BSA	1.7 ± 0.1
BSA + GO (10 mM)	28.2 ± 1.9
BSA + MGO (10 mM)	24.4 ± 2.2
BSA + DA (10 mM)	23.9 ± 1.2

The data are an average of triplicate data from two independent essays, and the standard deviations are given.

**Table 2:** Peptide-AGEs identified by LC-ESI-MS/MS ion trap analysis in gastric or gastrointestinal digests of BSA glycated with 1 mM GO or MGO.

No.	Identified Mass (Da)	Theoretical Mass (Da)	Sequence	Modification	Origin
Gastric digested peptides					
1	1151.572	1151.586	PKAFDEK(508)LF	CML	BSA + GO
2	991.037	991.465	LYYANK(163)Y	CML	BSA + GO
3	940.531	940.516	LILNR(462)LC	G-H1	BSA + GO
4	770.343	770.408	WSVAR(221)L	G-H1	BSA + GO
5	666.545	666.443	VLLR(351)L	MG-H1	BSA + MGO
6	784.404	784.423	WSVAR(221)L	MG-H1	BSA + MGO
7	1396.587	1396.714	YGFQNALIVR(413)Y	MG-H1	BSA + MGO
8	1685.861	1685.868	IARR(148)HPYFYAPEL	MG-H1	BSA + MGO
Gastrointestinal digested peptides					
9	1926.005	1925.962	VEK(298)DAIPENLPPLTADF	CML	BSA + GO
10	1099.576	1099.660	QEAK(351)DAFLG	CEL	BSA + MGO
11	1122.581	1122.712	AVSVLLR(351)LAK	MG-H1	BSA + MGO
12	1284.632	1284.704	VEVSR(431)SLGKVGT	MG-H1	BSA + MGO
13	729.470	729.366	VEVSR(431)S	MG-H1	BSA + MGO
14	632.342	632.292	FGER(212)A	MG-H1	BSA + MGO
